# Craniofacial Brown Tumor in Patients with Secondary Hyperparathyroidism to Chronic Renal Failure: Report of Two Cases in Cipto Mangunkusumo Hospital

**DOI:** 10.1155/2018/1801652

**Published:** 2018-09-12

**Authors:** Diani Kartini, Maria K. Siswiandari, Gunawan Wibisana, Erwin D. Yulian, Ahmad Kurnia, Sonar S. Panigoro, Azdi Z. Albar, Muchlis Ramli

**Affiliations:** Oncology Division, Department of Surgery, Dr. Cipto Mangunkusumo General Hospital, Faculty of Medicine, Universitas Indonesia, Jakarta, Indonesia

## Abstract

Brown tumor is a bone lesion that arises in the setting of excess osteoclast activity in hyperparathyroidism. It consists of fibrous tissue, woven bone, and supporting vasculature, while contains no matrix. The characteristic of brown-colored lesion is a result of hemosiderin deposition into the osteolytic cysts. Two cases of young women aged 26 and 29 years old, respectively, are known with a history of end-stage renal disease (ESRD). Dialysis is performed two times/week over the last 7 years. Our patients presented with an intraoral mass of the hard palate since 12 months ago and decreased body height of 10 cm. The lesion causes difficulties in swallowing and talking. Laboratory workup showed elevated parathormone or PTH (3.391 pg/mL and >5.000 pg/mL). Neck ultrasound showed enlargement of the parathyroid glands. Supporting examination to diagnose brown tumor are neck ultrasound, CT of the neck, and parathyroid sestamibi scan. We performed parathyroidectomy. Pathology revealed hyperplasia of the parathyroid. The tumor regressed significantly within 2 weeks following the surgery, and we still observe tumor regression as well as reduction in PTH level. As clinicians, we should be alert to other possible causes of bony lesions. Clinical examination, laboratory finding, and imaging present important information to diagnose brown tumor.

## 1. Introduction

Secondary hyperparathyroidism is a complication of renal failure, developed from many factors such as calcitriol deficiency, vitamin D deficiency, and the increased retention of phosphorus [1, 2]. These conditions cause hypocalcemia and trigger the production of PTH and hyperplasia of the parathyroid gland. The increase of fibroblast growth factor 23 (FGF-23) from renal failure also has a role in the development of secondary hyperparathyroidism [3]. The impacts of secondary hyperparathyroidism are bone resorption caused by the increased osteoclast activity, soft tissue calcification (calciphylaxis), cardiovascular events, pruritus, calcific uremic arteriolopathy, and many other [4].

Another complication of hyperparathyroidism is brown tumor, with incidence rate of 0.1%. Brown tumor is a giant cell granuloma which appears as a result of the imbalanced osteoclast activity and peritrabecular fibrosis [5]. It consists of fibrous tissue, woven bone, and supporting vasculature, while contains no matrix. Brown tumor histologically appears similar to giant cell tumor [6, 7]. The characteristic of brown-colored lesion is as a result of hemosiderin deposition into the osteolytic cysts [7]. This lesion rarely involves the craniofacial region, while it more commonly occurs in the mandible than in the maxilla [5]. It is also common in the long bones, ribs, and the pelvis. The lesion could be located in any part of the bones [8, 9].

Brown tumor presents as a fixated growing mass with bony consistency. The tumor itself is usually asymptomatic but causes disability in chewing, talking, and breathing if it occurs in the craniofacial region [8]. It may also cause secondary back pain or acute spinal compression if it occurs in the spinal cord [7]. Radiographic findings present the single or multiple osteolytic (radiolucent) lesion as well as the decreased density of the bones and a change in normal trabecular pattern [6].

### 1.1. Case 1

A 26-year-old female patient complained a growing mass in the hard palate since 12 months ago. This mass causes swallowing and closing mouth difficulty. She also complained about changes in the shape of her finger bones, mandible, and ribs. The decrease in height of 9 cm and weight loss of 10 kg were observed within 2 years. The patient experienced end stage of renal failure and has been treated with hemodialysis 2 times per week for seven years since 2009.

**Figure 1:**
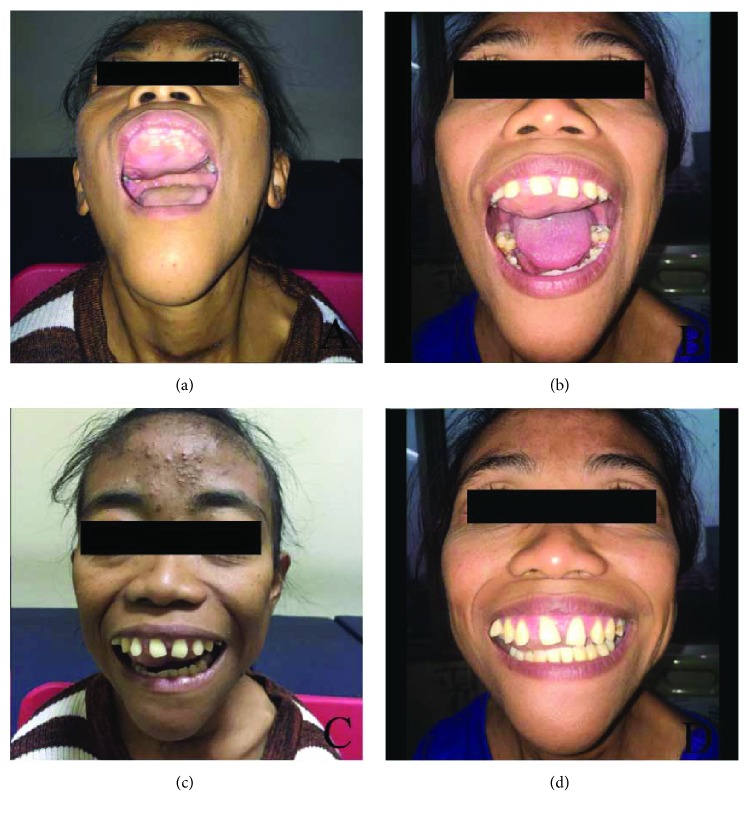
Case 1. (a) Hard palate mass. (b) Facial asymmetry due to the mass. (c, d) Regressed mass after parathyroidectomy.

The physical examination showed a mass in the palatum durum and mandible, measured 4 × 6 × 2 cm and 3 × 4 cm in size, respectively. The mass has rough surface, hard-solid consistency, with no tenderness. A mass in the right neck was measured 3 × 5 cm in size. The mass has smooth surface and solid consistency (Figure 1).

**Figure 2:**
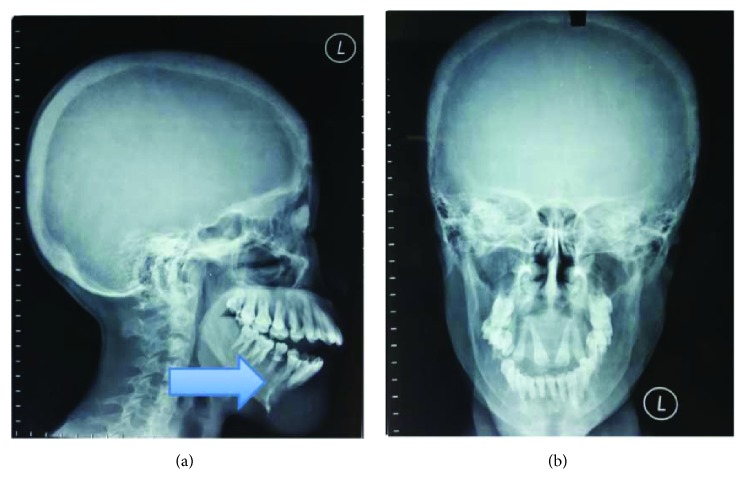
Case 1. (a) Bone survey radiographic examination of the cranium. (b) Multiple lytic lesion with well-defined border in the cranium. Arrow shows bone resorption activity in lower alveolar area.

**Figure 3:**
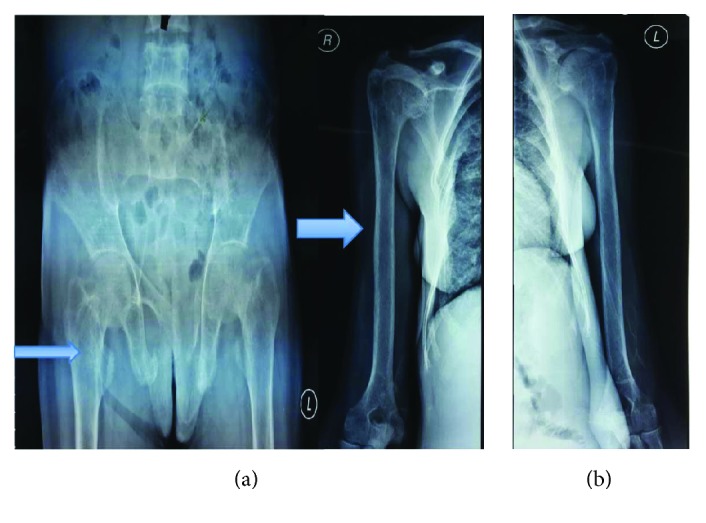
Case 1. Bone survey radiographic examination of the (a) pelvis showing multiple lytic lesion along the bilateral iliac bone and pubic bone and the (b) humerus and clavicle showing multiple lytic lesion along the lateral edge of the humerus. Pathologic fractures of the distal part of the left clavicle are observed, increased bone resorption among the bilateral clavicle.

**Figure 4:**
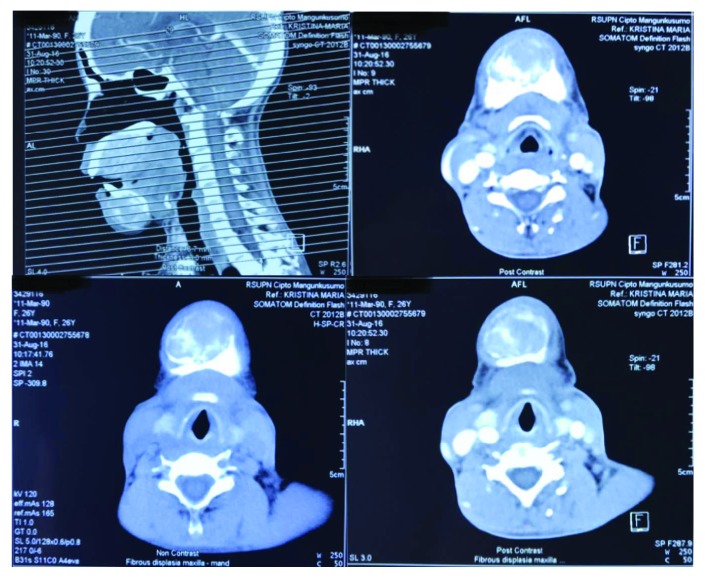
Case 1. Paranasal CT scan. Decreased diffuse with ground glass appearance among the cranium, facial, and cervicothoracic bones. Multiple lytic lesion in the maxillary bones.

The result of the palatum and mandible biopsy was compatible with ossifying fibroma or fibrous dysplasia. Neck ultrasonography showed a solid mass in the right thyroid, bilateral cystic nodule in the thyroid lobes, and small cystic nodule in the thyroid isthmus. Bone survey showed the diffused decreasing bone density, fractured right clavicle, multiple lytic lesions among the cranium, humerus, clavicle, iliac, and pubic bone (Figure 2 and Figure 3). Paranasal sinus CT showed a decreased bone density in the cranial, facial, and cervicothoracic bones; lytic lesion in the maxilla bone extended to the palatum durum and shifted the tongue inferiorly, with the measurement of 5.2 × 4.6 × 3.7 cm (Figure 4). Similar lytic lesions were also found in the midline and right mandible, measured 3.8 × 3.2 × 2.8 and 2 × 2.3 × 2.1 in size, respectively. Workup laboratory examination showed the elevated intact parathyroid hormone level (PTH) (3.391 pg/mL), ureum (124 mg/dL), and creatinine (6.60 mg/dL) and normal range of calcium level (9.5 mg/dL) and phosphate (3.6 mg/dL). Histopathologic findings suggest ossifying fibroma with a differential diagnosis of fibrous dysplasia. No signs of malignancies were revealed in the histopathologic examination.

Based on the physical examination and laboratory findings, we concluded brown tumor as the diagnosis due to secondary hyperparathyroidism. The treatment for this patient was right parathyroidectomy and right isthmulobectomy with frozen section. The right isthmulobectomy was chosen because the patient has secondary diagnosis which was right nontoxic nodular enlargement of the thyroid gland. In addition, the technique was considered to be appropriate to access the parathyroid gland with the antecedent removal of the thyroid gland. Frozen section revealed hyperplasia of the right parathyroid. Three days following the surgery, PTH level was significantly decreased from the initial preoperative level (851.10 pg/mL). Result from definitive pathology anatomy was hyperplasia of the parathyroid gland.

One month following the surgery, the patient felt regression among her hard palate and mandible mass and ease in swallowing. During follow-up, we performed additional evaluative examination. Neck 4D CT was done 2 times at the 2nd and 5th month of follow-up. The first one showed multiple lymphadenopathy around the neck and left supraclavicle, no residual mass in the right thyroid, no abnormality in the left thyroid, and no ectopic parathyroid gland was found. While the second Neck 4D CT found a single solid lesion in the left thyroid and 2 solid lesions in aortopulmonary window, suggestive of parathyroid nodes. At the 6th month of follow-up, sestamibi parathyroid scan showed no abnormality and no residual mass in the right thyroid lobe. PTH level evaluation performed in the 4th and 7th month showed an elevation to 909.7 pg/mL and 1.137 pg/mL, respectively.

Patient complained pain in all her bones and started walking with a cane. Seven months following the first surgery, we decided to perform left parathyroidectomy and left lobectomy with frozen section and intraoperative PTH evaluation. Thirty minutes following parathyroidectomy, the PTH level decreased significantly to 76.95 pg/mL. Frozen section result revealed hyperplasia of the left parathyroid.

Two weeks following the second surgery, the mass regressed significantly, and symptoms were much reduced.

### 1.2. Case 2

**Figure 5:**
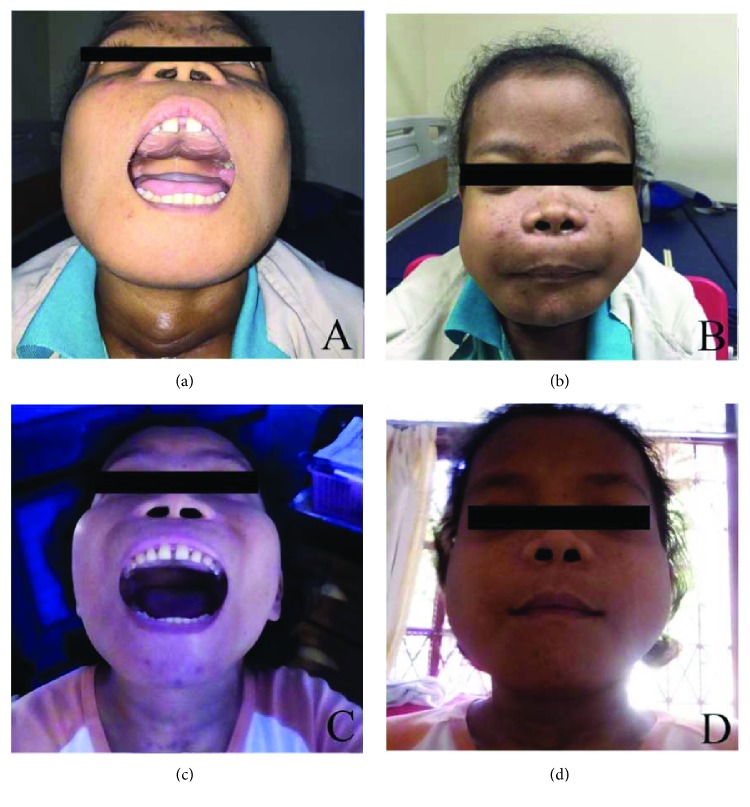
Case 2. (a) Hard palate mass. (b) Swelling in the bilateral maxilla and mandible. (c, d): Regressed mass after parathyroidectomy.

A 31-year-old female patient complained a mass in her maxilla and mandible. The mass was gradually increasing in size over the last 6 months. The mass caused talking disturbances, swallowing difficulty, dilating gap between upper teeth, and narrowing gap between lower teeth. She also complained hard masses in her fingers and the middle of chest. There was a decrease in height (15 cm in 8 years) and weight (19 kg in 8 years). The patient was on end stage of renal failure and has been treated with hemodialysis 2 times per week for eight years since 2008. The patient was planned to be performed renal transplantation. Comorbidities were reported among this patient, which are chronic heart failure, grade II hypertension, lung tuberculosis on therapy, and hepatitis C. The physical examination showed facial asymmetry with mass in the palatum durum, measured 4 × 3 cm in size (Figure 5). Palpation revealed hard consistency originated from the bone, smooth surface, and no tenderness.

**Figure 6:**
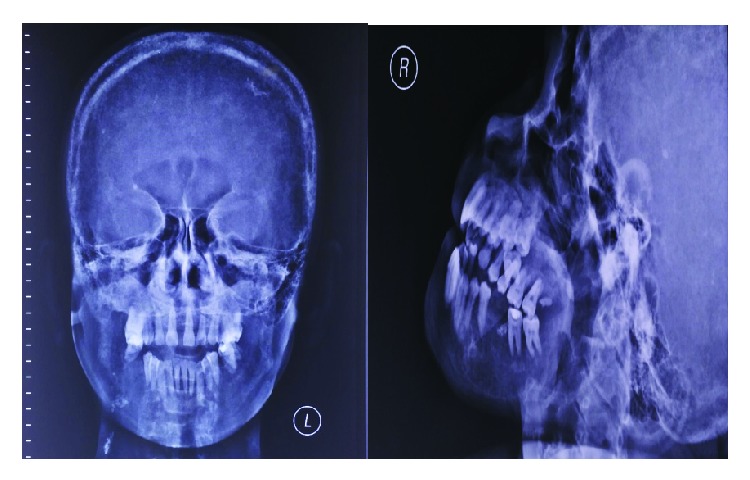
Case 2. Bone radiographic examination of the skull showing multiple lytic lesion among the calvaria, decreased diffuse bone density among the calvaria, and multiple lytic lesion in the calvaria, maxilla, and mandible. Salt and pepper appearance in the calvaria.

**Figure 7:**
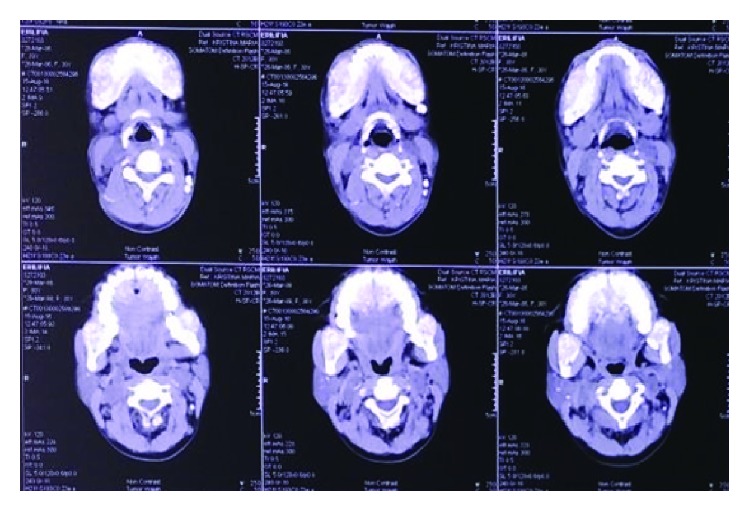
Case 2. Head CT scan showing diffused hyperostosis with multiple lytic lesion among the bones of maxilla, mandible, and the skull.

X-ray of the maxilla and mandible showed diffused decreasing bone density and multiple lytic lesion, which suggest metabolic bone disease or renal osteodystrophy or hyperparathyroidism (Figure 6). BNO showed compression fracture in thoracolumbar vertebrae and multiple calcification in soft tissue abdominal and pelvic region compatible with renal osteodystrophy. Bone survey also showed the decreased bone density. Facial CT with contrast showed diffuse hyperostosis, lytic lesion in the mandible bone, maxillary bone, cranium, and multiple calcification in the soft tissue which is compatible with osteorenal dystrophy (Figure 7). Thyroid ultrasonography showed bilateral multiple cysts in the thyroid, a firm lesion with calcification in the left parathyroid fossa, and oval lesion in the left pericarotid. Parathyroid scan showed an increased activity in the inferior aspect of left thyroid which was suggestive of parathyroid adenoma.

Laboratory examination showed the elevated ureum (202 mg/dL), creatinine (11.1 mg/dL), and normal calcium level (9.7 mg/dL). In November 2016, PTH level was >5000 pg/mL and phosphate level was 6.1 mg/dL.

Physical examination and laboratory examination lead us to brown tumor as the diagnosis due to secondary hyperparathyroidism. The treatment for this patient was subtotal parathyroidectomy with frozen section and intraoperative PTH evaluation. Intraoperative parathyroid hormone level was evaluated following subtotal parathyroidectomy. The result decreased to normal range 10.37 pg/mL (normal range: 10–65 pg/mL). Frozen section revealed hyperplasia of the parathyroid. Calcium level was evaluated every day for 7 days, and the result ranged from 5.0 mg/dL to 7.4 mg/dL. The parathyroid hormone on the 3rd month following the surgery was 4.31 pg/mL. Definitive pathology anatomy result revealed hyperplasia of the parathyroid with water clear cell parathyroid adenoma.

The masses in the maxilla, mandible, and sternum were observed to be significantly regressed. This patient then underwent renal transplantation approximately 4 months following parathyroidectomy. One month following the renal transplantation, PTH level was 6 pg/mL.

## 2. Discussion

There are numerous options for hyperparathyroidism treatment such as dietary modification, pharmacological therapy (e.g., vitamin D receptor activator, non-calcium-based phosphate binders, calcimimetics, and paricalcitol), and parathyroidectomy [10]. The indication for surgical intervention are PTH level > 800 pg/mL, hypercalcemia, hyperphosphataemia, hypercalciuria, and severe symptom of hyperparathyroidism including unbearable bone pain, osteoporosis/osteopenia, and severe pruritus [11, 12]. Failure to maintain normal level of phosphate, calcium, and PTH among patients with chronic kidney disease promotes to the higher mortality rate due to cardiovascular complication related to ectopic calcification [13]. Therefore, the parathyroid intervention should be considered to replace pharmacological treatment [14].

It is very important to determine the extent of parathyroidectomy prior to surgery to prevent recurrent or persistent disease and avoidance of permanent hypoparathyroidism [12, 15]. Therefore, there is a need for further investigations such as neck ultrasound, sestamibi scan, CT imaging, 4D CT, and MRI [15, 16]. Selection of imaging depends on surgeons' preference and the available facilities.

The first choices for preoperative examination are combination of ultrasonography and technetium Tc99m sestamibi scan, with the sensitivity of 74%–95% for single gland disease. Whereas for multigland disease and multiple adenomata, the sensitivity of ultrasonography is 15%–35% and sestamibi scan is 30%–44% [17]. 4D CT may be useful for preoperative evaluation in patients with multigland disease, reoperative parathyroid patients, patients with mild hypercalcemia, and patients with negative or inconclusive ultrasound and sestamibi scan results [16]. With sensitivity ranges from 85.7 to 89.4% for localization, 4D CT provides greater sensitivity compared with ultrasonography and sestamibi scan and able to predict multigland disease (85.7%) [16, 18, 19]. The localization result from 4D CT would be superior if combined with intraoperative parathyroid measurement [20].

There are three methods of surgical treatment: subtotal parathyroidectomy, total parathyroidectomy with autotransplantation, and total parathyroidectomy without autotransplantation [10]. Selection of surgical method may be based on the expected survival after procedure and surgeon's preference. Subtotal parathyroidectomy is suitable for dialysis patients with kidney transplantation and younger patients, while total parathyroidectomy with autotransplantation is recommended for dialysis patients without kidney transplantation. Total parathyroidectomy without autotransplantation is recommended for enlarged parathyroid glands revealed at surgery or reoperation patient [21].

In the first case, we decided to perform right parathyroidectomy and right isthmulobectomy without intraoperative PTH examination due to limited facilities. The isthmulobectomy was chosen because the patient has secondary diagnosis which was nontoxic nodular enlargement of the thyroid gland. In addition, the technique was considered to be appropriate to access the parathyroid gland with the antecedent removal of the thyroid gland.

In the 4th and 7th month of follow-up, it is found that the PTH level was still high, 909.7 pg/mL and 1.137 pg/mL, respectively. 4D CT revealed single solid lesion in the left thyroid and 2 solid lesions in the aortopulmonary window, suggestive of parathyroid nodes. Therefore, 7 months after the first operation, we performed excision of superior part of the left parathyroid and left lobectomy with intraoperative PTH examination, and the result was 76.95 pg/mL. In the second case, we performed subtotal parathyroidectomy with intraoperative PTH examination, and the result was 10.37 pg/mL. In the 3rd month of follow-up, PTH level was 4.31 pg/mL. This patient underwent renal transplantation approximately 7 months after subtotal parathyroidectomy. PTH level one month after follow-up was 6 pg/mL.

Our delay in the PTH examination is due to the fact that this examination is not covered by the national health insurance. PTH examination is considerably expensive in Indonesia, which costs approximately 60 USD. Therefore, the PTH examination is not a routine test for this patient.

## 3. Conclusion

Brown tumor is one of the escalating problems due to secondary hyperparathyroidism because of ESRD. The clinical presentation of secondary hyperparathyroidism should be integrally assessed to reveal the possibility of developing brown tumor in the future, especially for patients who present with mass in the maxilla and mandible. A significant increase in the level of PTH and decreased bone density lead to the diagnosis of brown tumor. 4D CT and sestamibi are suggested to be the preferable examination to reveal the parathyroid abnormalities. Surgery and intraoperative assessment of PTH are the suggested treatment of choice.
